# ﻿Two new species of *Rubus* L. (Rosaceae) from the western Andes of Ecuador

**DOI:** 10.3897/phytokeys.187.76963

**Published:** 2021-12-20

**Authors:** David A. Espinel-Ortiz, Katya Romoleroux

**Affiliations:** 1 Laboratorio de Botánica Sistemática, Herbario QCA, Facultad de Ciencias Exactas y Naturales, Pontificia Universidad Católica del Ecuador, Av. 12 de Octubre 1076 y Vicente Ramón Roca, 170525 Quito, Ecuador Pontificia Universidad Católica del Ecuador Quito Ecuador

**Keywords:** Andean slopes, blackberry, Chocó, Ecuadorian, Rubeae, taxonomy

## Abstract

Two new species of *Rubus* (Rosaceae) from the western Andes of northern Ecuador are described. *Rubuslongistipularis* D.A. Espinel-Ortiz & Romol. is a scandent or climbing shrub found in the mountain forests of Chocó Andino from northern Ecuador. *Rubusmaquipucunensis* D.A. Espinel-Ortiz & Romol. is a vine or climbing shrub found in the rainforests of Chocó Andino from Pichincha and Santo Domingo de los Tsáchilas. The species mentioned here are morphologically differentiated from all the *Rubus* species from Ecuador with a detailed botanical description, illustrations and photographs. We also report, for the first time, possible hybridisation between *R.longistipularis* and *R.boliviensis* Focke, as the samples reviewed showed mixed characteristics from both species.

## ﻿Introduction

*Rubus* L. is the most diverse genus of the Rosaceae family with more than 500 species classified in 14 subgenera (Focke 1910, 1911, 1914; Kalkman 1987; Jarvis 1992; Bean 1997). The genus presents a cosmopolitan distribution with most species in Asia, Europe and North America (Lingdi and Boufford 2003). Recently, Carter et al. (2019) suggested that the genus originated in North America, and migrated repeatedly to the other continents, thus explaining that these events may have favoured the high diversity of this genus in Asia, Europe and the world. In South America, only four subgenera with fewer than 50 species have been reported, mostly throughout the Andes (Romoleroux 1996; Romoleroux et al. 2014; Espinel-Ortiz and Romoleroux 2020).

In Ecuador, the genus *Rubus* inhabits most of the Andean ecosystems from 450 to 4500 m a.s.l. However, it is most abundant from 2300 to 3500 m a.s.l., especially in disturbed areas, in hedges, clearings and on margins of cloud forests, and in “páramo” regions (Espinel-Ortiz and Romoleroux 2020). Here, 22 species of *Rubus* have been recorded and classified in three subgenera: *Idaeobatus* (Focke) Focke, *Orobatus* Focke and RubusL. The introduced subgenus Idaeobatus comprises 3 species, while the native subgenus Rubus consists of 10 native species, and Andean endemic subgenus Orobatus entails 7 native and 2 endemic species in the country (Romoleroux 1996; Espinel-Ortiz and Romoleroux 2020). The species described here belong to the subgenus Rubus, and both are distributed on the western Andean slopes of northern Ecuador. Herbarium specimens representing these species showed low sample numbers and were often annotated as *Rubusboliviensis*, *Rubusglaucus* and *Rubusfloribundus* because of the resemblance to these species. However, vegetative and reproductive characters of the new species differ greatly from those of the species reported by Romoleroux (1996).

## ﻿Methodology

*Rubus* collections of Herbaria HUTI, Q, QAP, QCA and QCNE were revised, and samples not fitting with the species reported for Ecuador were studied. Additional samples from AAU, DAV, HA and MO were revised from online images.

The botanical terms used in the descriptions followed those used by Stearn (1986), and the pubescence types were based on the terms of Hickey and King (2000), and Wilhelm and Rericha (2020). Some specimens examined for the descriptions (e.g. D. Espinel-Ortiz & H.G. Abad 281) were mounted in more than one herbarium sheet, and/or have additional dry or alcohol material, and each part had its own herbarium barcode (DAV, QAP and QCA). For these samples, we wrote all the herbarium barcodes for each part in type and examined specimens when available.

The geographic coordinate from sample P. Delprete & G. Webster 6073 (QCA-240552) was misplaced, since the sample was collected in Ecological Reserve Maquipucuna (Pichincha) and the coordinate of the label was from Ecological Reserve Cotacachi-Cayapas (Esmeraldas), approximately 42 km to the north. Therefore, the coordinate was eliminated in the examined specimens.

## ﻿Taxonomic treatment

### ﻿ Rubus longistipularis

Taxon classificationPlantaeRosalesRosaceae

Espinel-Ortiz & Romol. sp. nov.

FA31F32D-A2FF-529F-B8D9-330ED001952E

urn:lsid:ipni.org:names:77234524-1

Figs 1 , 2 , 3

#### Diagnosis.

*Rubuslongistipularis* is characterised by its villous to pannose white pubescence in branches, stipules, petioles, and leaves, and pannose and sericeous pubescence in sepals, its long (20.0–34.7 mm) stipules, 15–27 secondary veins on leaflets, flowers with deeply concave, pink petals with fuchsia borders, and fruits with up to 195 small drupelets (1.5–3.1 × 0.9–2.5 mm).

**Figure 1.**
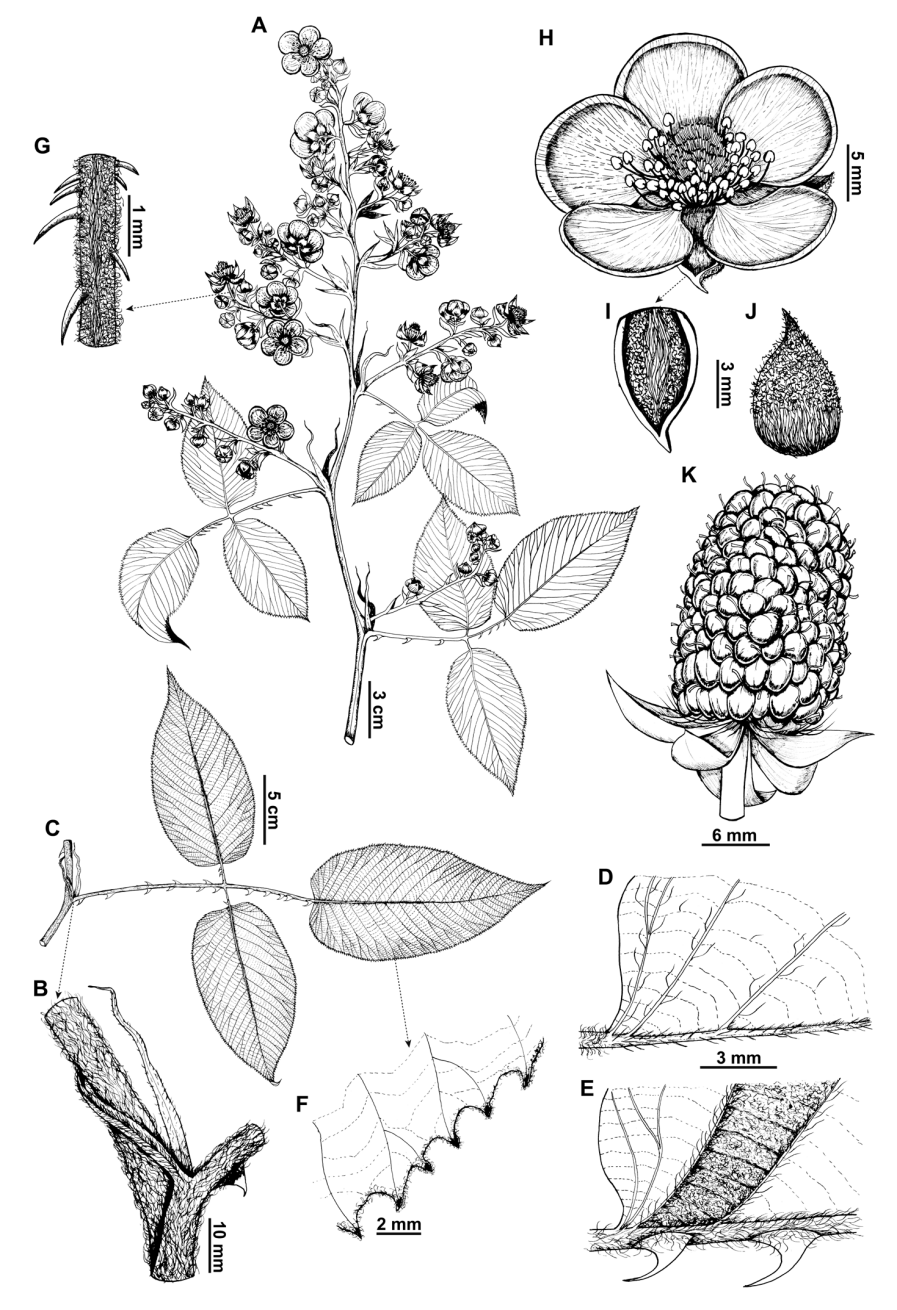
*Rubuslongistipularis* D.A. Espinel-Ortiz & Romol. **A** habit and inflorescence **B** branch and stipule **C** leaf **D** leaf adaxial surface **E** leaf abaxial surface **F** leaf border **G** pedicel **H** flower **I** sepal adaxial surface **J** sepal abaxial surface **K** fruit. (**A** based on P.M. Jørgensen & S.S. Vire 56085 (QCA) **B–K** based on D. Espinel-Ortiz & H.G. Abad 281 (QCA)). Illustrations by Carla Rodríguez.

#### Type.

**Ecuador. Pichincha**: Nono-Tandayapa road, between km 116–117, 00°01.787'S, 78°38.567'W, 1950 m, 26 Jul 2021 (fl, fr), *D. Espinel-Ortiz & H.G. Abad 281* (holotype: QCA (QCA-243418 and QCA-7010714 to QCA-7010723); isotypes: HA, HUTI, QAP).

**Figure 2.**
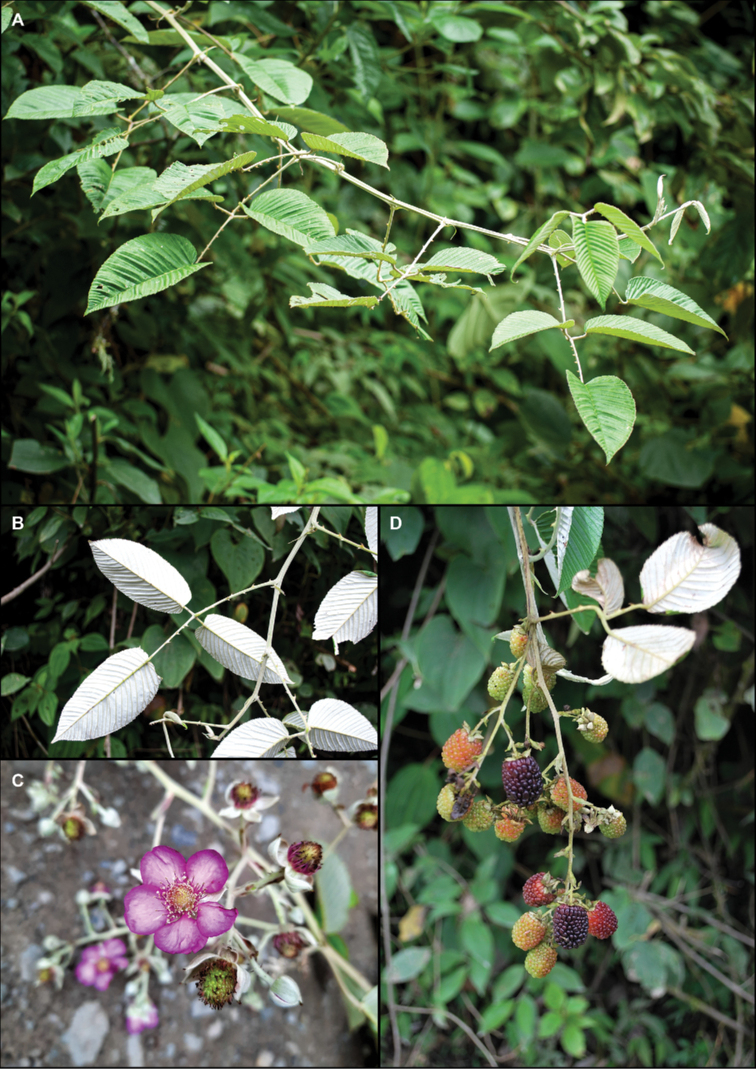
*Rubuslongistipularis* D.A. Espinel-Ortiz & Romol. **A** habit **B** leaf abaxial surface and stipules **C** flower **D** infructescence with immature and mature fruits. Photos by David A. Espinel-Ortiz.

#### Description.

**Scandent or climbing shrub**, growing up to more than 3 m over the vegetation, glaucous pubescence all over the plant, with all prickles, from the base ⅓–⅔ villous, and glabrous towards the apex, rarely with subsessile glands. **Branches** obtuse-angled, woody, light greenish-white when young to dark brown when old, villous to pannose, 3.7–7.2 mm diam., with scattered sessile and subsessile glands; sparsely prickly, unarmed or with up to 4 prickles (per total area of 5 cm long of the branch), falcate, 2.6–5.3 × 3.1–7.9 mm. **Stipules** asymmetrically, anguste subulate, (14.7–) 20.0–34.7 × 1.7–3.3 mm, margin entire, chartaceous; adaxial surface villous on the midvein and towards the margin, rarely with subsessile glands towards the margin; abaxial surface sparsely pannose to villous with scattered subsessile glands. **Petioles** (3.96–) 9.61–12.2 cm long, villous to pannose with scattered long hairs, especially towards the leaf blade or when young, with (1–) 3–8 (–12) prickles, falcate, 2.0–4.5 × 2.0–5.8 mm; lateral petiolules (2.5–) 6.4–14.7 (16.3–) mm long, unarmed or with up to 4 prickles, falcate, 1.2–1.9 × 1.7–3.0 mm; terminal petiolules (1.0–) 2.4–8.3 cm long, with (2–) 5–15 prickles, falcate, 1.5–3.1 × 1.7–5.4 mm. **Leaves** trifoliate, rarely 4–5-foliolate; leaflets ovate to elliptic, base rounded to obtuse, or subcordate, apex acuminate to slightly apiculate, margin serrulate, lateral leaflets (4.5–) 6.4–14.4 × (2.5–) 3.6–7.9 cm, terminal leaflet (5.9–) 8.5–17.0 × (3.2–) 4.2–9.6 cm, chartaceous, with (11–) 15–27 secondary veins; adaxial surface sparsely hirsute on the midvein, and sparsely pilose mainly on secondary veins and slightly tomentose towards the border, with scattered orange to red sessile and subsessile glands, unarmed; abaxial surface sparsely villous on the midvein and secondary veins, and pannose, with scattered orange to red subsessile glands on the veins, and 2–18 prickles on the primary vein, falcate, 0.2–2.5 × 0.5–3.6 mm. **Inflorescences** lax, compound, terminal and axillary cymes, 23–106-flowered, 9.6–32.5 cm long, with simple or trifoliate leaves below; peduncles terete, white to slightly brownish, (9.5–) 14.2–66.1 (–81.0) mm long, pannose, eglandular, unarmed or with up to 3 prickles, falcate, 0.5–1.7 × 0.8–2.3 mm; pedicels terete, white, pannose and slightly sericeous, 5.6–16.8 (–23.3) mm long, eglandular, unarmed or with up to 14 prickles, triangular to falcate, 0.2–1.5 × 0.1–1.8 mm. **Flowers** 17.5–24.0 mm diam.; sepals 5, ovate to elliptic, apex acuminate, margin involute, 8.8–11.9 × 4.0–5.8 mm, light greenish-grey to greenish-white, acrescent; adaxial surface deeply concave, sericeous, and pannose towards the apex and the margin, eglandular, unarmed; abaxial surface deeply convex, shortly lanate and slightly tomentose towards the apex, eglandular, unarmed; petals 5, broadly elliptic to broadly obovate, margin entire, 8.8–12.7 × 9.8–12.6 mm, fuchsia when opening, completely pink or pink with fuchsia borders when fully opened, glabrous, eglandular, adaxial surface deeply concave, abaxial surface deeply convex; stamens with anthers glabrous, filaments fuchsia, glabrous; pistils, stigmas and styles glabrous, ovaries densely villous. **Fruits** green to dark red when immature, and black at maturity, ovoid to oval, 11.5–23.9 × 8.0–17.1 mm (when fresh); drupelets 50–195 per receptacle, 1.5–3.1 × 0.9–2.5 mm (when fresh), sparsely villous and deeply villous towards the apex.

**Figure 3.**
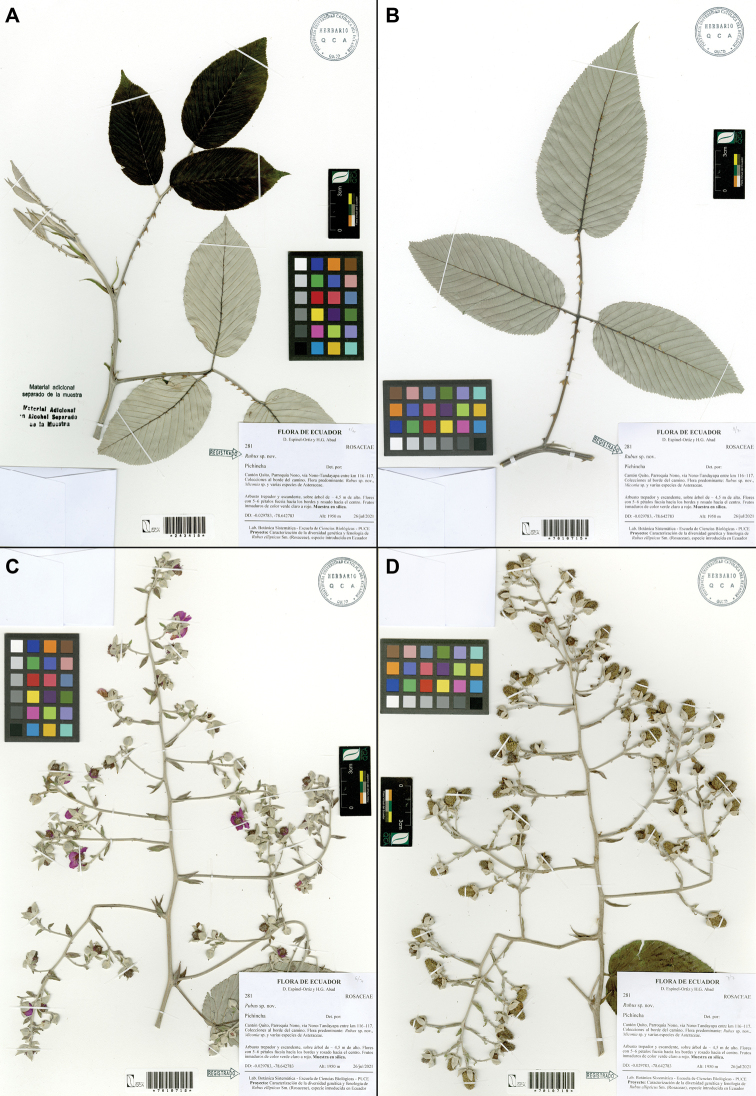
*Rubuslongistipularis* D.A. Espinel-Ortiz & Romol. Holotype collection D. Espinel-Ortiz & H.G. Abad 281 (QCA) **A** branch apex QCA243418 **B** leaf abaxial surface QCA7010715 **C** terminal inflorescence QCA7010718 **D** infructescence QCA7010719.

#### Additional specimens examined (Paratypes).

**Ecuador. — Santo Domingo de los Tsáchilas**: Chillogallo-Santo Domingo road, below Chiriboga, 00°15.000'S, 78°47.000'W, 2000 m, 13 Aug 1980 (fl), *L. Holm-Nielsen*, *B.* Øllgaard *& C. Sperling 24755* (AAU). – **Imbabura**: Cuicocha-Apuela road, km 28, disturbed cloud forest, 00°22.000'N, 78°28.000'W, 2480–2670 m, 05 Oct 1984 (fl), *P.M. Jørgensen* & *S.S. Vire 56085* (AAU, QCA (QCA-91776), QCNE (QCNE-12185)). – **Pichincha**: Quito, west side from Pelagallo, sendero Guantopungo-Chichipunta trail, 00°04.400'N, 78°34.470'W, 2432 m, 25 Sep 2021 (fl, fr), *C.E. Cerón*, *C.I. Reyes 89354* (QAP (QAP-107614 and QAP-107574)); Quito, Nanegalito, Golán, road between Edén Mágico and Ecological Reserve San Luis, 00°04.460'N, 78°33.340'W, 2300–2500 m, 06 Feb 2021 (fr), *C.E. Cerón*, *C.I. Reyes & C. Bacuilima 87651* (QAP (QAP-106251), QCA (QCA-243453)); Quito, Nanegalito, Golán, near Mrs Margarita Bacuilima property, 00°05.370'N, 78°33.420'W, 2281 m, 26 Apr 2021, *C.E. Cerón*, *C.I. Reyes & J. Bacuilima 88206* (QAP (QAP-106667)); Quito, Nanegalito, Golán, road between Edén Mágico-El Alí, 00°06.260'N, 78°33.140'W, 2603 m, 18 May 2021, *C.E. Cerón*, *C.I. Reyes & C. Bacuilima 88386* (QAP (QAP-105946)); Quito, Nanegalito, El Porvenir, near Guerrero family property, 00°06.190'N, 78°33.240'W, 2427–2500 m, 21 Aug 2021 (fr, fl), *C.E. Cerón*, *C.I. Reyes y J. Bacuilima 89113* (QAP (QAP-107423), QCA (QCA-243441), QCNE); Orchid Reserve Pahuma, Chorrera trail, 00°01.000'N, 78°38.000'W, 2000–2500 m, 08 Sep 1996 (fl), *C.E. Cerón & E. Freire 32387* (QAP (QAP-25420)); Tandayapa-Tambo-Nono road, disturbed cloud forest, 00°01.429'S, 78°38.630'W, 1974 m, 05 Mar 2021, *D. Espinel-Ortiz & C. Restrepo 276* (QCA (QCA-243396, QCA-7010701 and QCA-7010702)); Nono-Tandayapa road, between km 117–118, 00°01.967'S, 78°38.491'W, 1925 m, 29 Oct 2021 (fr), *D. Espinel-Ortiz & C. Restrepo 296* (QCA); Nono-Tandayapa road, between km 123–124, 00°02.533'S, 78°38.204'W, 2125 m, 02 Aug 2021 (fl, fr), *D. Espinel-Ortiz*, *C. Restrepo & A. Sanguano 285* (QCA (QCA-243397 and QCA-7010703)); same locality as for preceding, 00°02.539'S, 78°38.215'W, 2091 m, 21 Oct 2021 (fr), *D. Espinel-Ortiz & H.G. Abad 294* (HUTI, QAP, QCA (QCA-243454, QCA-7010752 and QCA-7010752), QCNE); same locality as for preceding, 00°02.525'S, 78°38.210'W, 2091 m, 21 Oct 2021 (fr), *D. Espinel-Ortiz & H.G. Abad 295* (QCA (QCA-243452)); Quito-Chiriboga road, 2 km after Corazón Station of Petroecuador, 00°16.814'S, 78°42.067'W, 2399 m, 13 Dec 2020, *D. Espinel-Ortiz*, *C. Restrepo & C. García 262* (QCA (QCA-243398, QCA-7010704 to QCA-7010709), QUSF); same locality as for preceding, 00°16.804'S, 78°42.144'W, alt. 2377 m, 13 Dec 2020, *D. Espinel-Ortiz*, *C. Restrepo & C. García 264* (LOJA, QCA (QCA-243390)); same locality as for preceding, 00°16.809'S, 78°42.142'W, 2355 m, 28 Jan 2021, *D. Espinel-Ortiz & C. Restrepo 268* (QCA (QCA-243395 and QCA-7010700)). – **Cotopaxi**: Campo Alegre, ca. 20 km NE of Sigchos, 00°35.050'S, 78°47.600'W, 2614 m, 11 Jul 2003 (fl, fr), *J. Ramos*, *L. Ramos*, *A. Tigse & R. Tigse 5801* (MO, QCA (QCA-137959), QCNE (QCNE-200267)).

#### Distribution.

**Figure 4.**
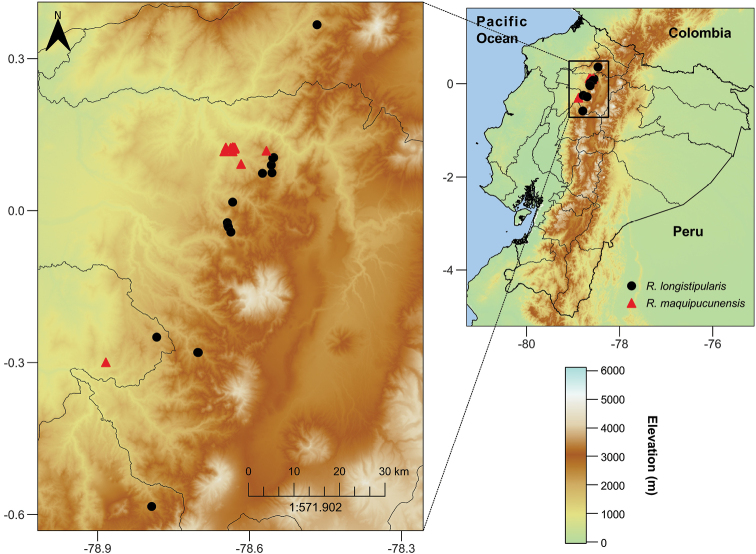
Distribution map of *Rubusmaquipucunensis* (red triangles), and *R.longistipularis* (black circles) from the western Andes of North Ecuador. Map generated by David A. Espinel-Ortiz.

*Rubuslongistipularis* is distributed in the north of the Ecuadorian Western-Cordillera from 1900 to 2700 m a.s.l., in the provinces of Santo Domingo de los Tsáchilas, Imbabura, Pichincha and Cotopaxi (Fig. 4).

#### Ecology.

This species occurs in Chocó Andino montane cloud forests dominated by trees and shrubs and also in nearby disturbed areas. *Rubuslongistipularis* can be found living in sympatry with *Rubusadenotrichos* Schltdl., *R.boliviensis* Focke, *R.glaucus* Benth., *R.niveus* Thunb. and *R.urticifolius* Poir. As branches grow older, they may become glabrescent and lose prickles and stipules. In some flowers, two sepals may be united in the apex, but they separate completely when fruiting occurs. Since flower blossoming, it takes about three months for the fruits to appear and ripen. Flowering and fruiting collections dated from the months of February, July, August, September and October.

#### Etymology.

The specific epithet refers to the long (20.0–34.7 mm) asymmetrically, anguste subulate stipules.

#### Preliminary assessment of conservation status.

*Rubuslongistipularis* is known from five localities, impacted by human activities, including regression to agriculture and road openings. Following the IUCN (2019) guidelines, based on the geographic distribution and altered land use at the localities, this species should be categorised as least concern (LC).

#### Notes.

*Rubuslongistipularis* may resemble *R.boliviensis* by its habit and big leaves, but differs from this species by its white villous to pannose branches, in contrast with the pannose, pilose or puberulent to glabrescent branches of *R.boliviensis*. Moreover, *R.longistipularis* has trifoliate leaves with ovate to elliptic leaflets while *R.boliviensis* has 5-foliolate leaves with ovate-elliptic leaflets. Furthermore, *R.longistipularis* has fruits with more (50–195) and narrower (1.5–3.1 × 0.9–2.5 mm) drupelets while *R.boliviensis* has fruits with fewer (20–50) and wider (2.0–3.0 × 2.0–3.0 mm) drupelets. *Rubuslongistipularis* resembles *R.glaucus* by its habit, trifoliate leaves and fruits, but differs by its white villous to pannose branches, pannose and slightly sericeous pedicels and bigger petals (8.8–12.7 × 9.8–12.6 mm) compared to the glabrous and pruinose branches, glabrous pedicels and smaller petals (7.0–10.0 × 5.0–8.0 mm) of the latter. Moreover, *R.longistipularis* differs from both species by its longer stipule (20.0–34.7 × 1.7–3.3 mm), in contrast with the smaller stipules of *R.boliviensis* (6.0–10.0 × 1.0–2.0 mm) and *R.glaucus* (5.0–12.0 × 0.3–0.8 mm).

### ﻿ Rubus maquipucunensis

Taxon classificationPlantaeRosalesRosaceae

Espinel-Ortiz & Romol. sp. nov.

48E1D568-6448-5B4D-AFFB-C861F4DE4B3A

urn:lsid:ipni.org:names:77234525-1

Figs 5 , 6 , 7

#### Diagnosis.

*Rubusmaquipucunensis* is characterised by its villous to villous-hispid branches, trifoliate leaves with broadly elliptic or broadly ovate to elliptic leaflets, long inflorescences (22.6–59.4 cm long), flowers with fuchsia or pink petals and fuchsia filaments, and fruits with big drupelets (4.0–6.1 × 3.1–5.4 mm).

**Figure 5.**
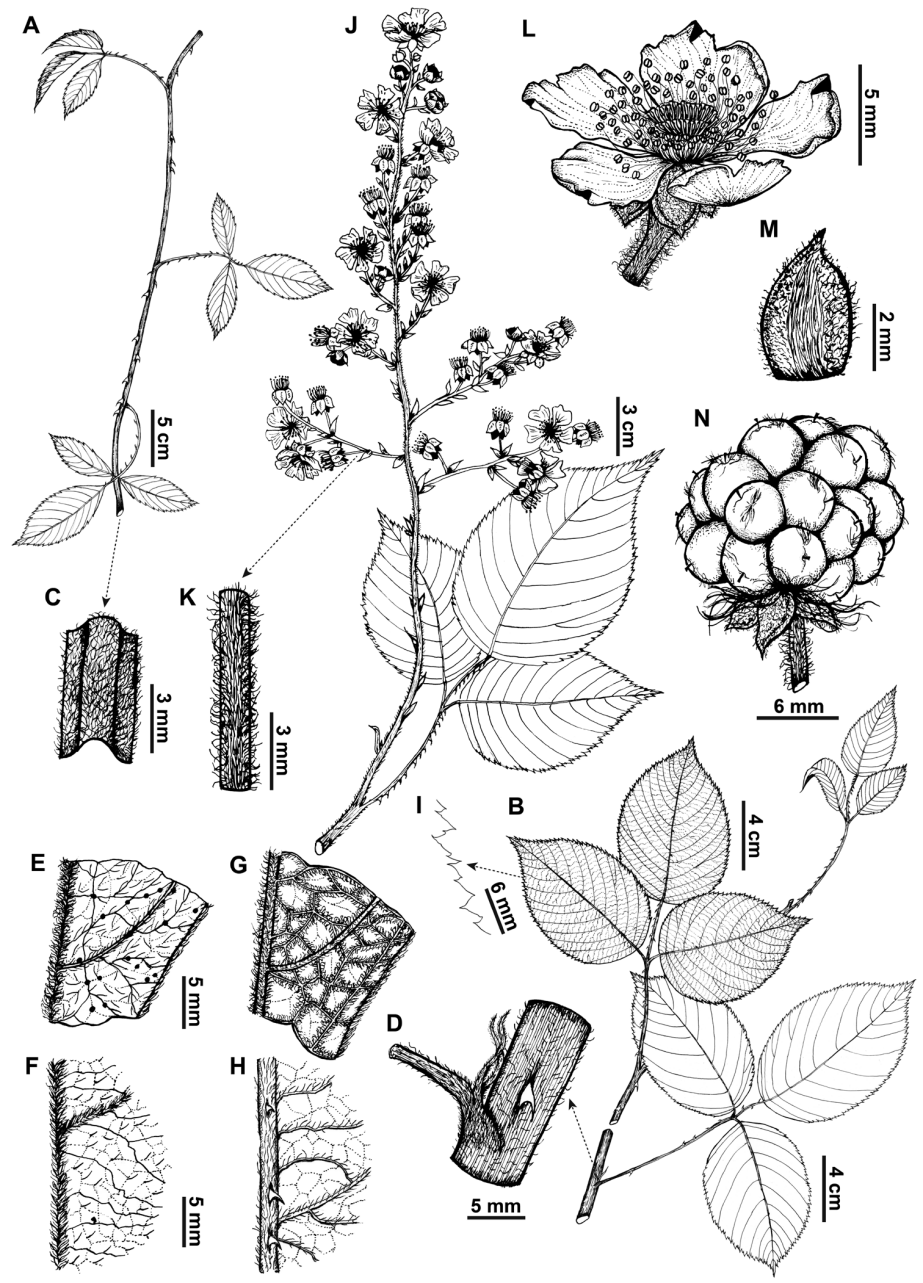
*Rubusmaquipucunensis* D.A. Espinel-Ortiz & Romol. **A** vine **B** scandent shrub **C** vine branch **D** shrub branch and stipule **E** vine leaf adaxial surface **F** shrub leaf adaxial surface **G** vine leaf abaxial surface **H** shrub abaxial surface **I** leaf border **J** inflorescence **K** pedicel **L** flower and sepal adaxial surface **M** sepal abaxial surface **N** fruit. (**A–I** based on D. Espinel-Ortiz, C. Restrepo & A. Sanguano 275 (QCA), **J** based on D. Espinel-Ortiz, C. Restrepo & A. Sanguano 269 (HUTI) **L–N** based on D. Espinel-Ortiz, C. Restrepo & A. Sanguano 269 (QCA)). Illustrations by Carla Rodríguez.

#### Type.

**Ecuador. Pichincha**: cantón Quito, parroquia Nanegal, in front of the Ecological Reserve Maquipucuna entrance, 00°07.457'S, 78°37.744'W, 1278 m, 11 Feb 2021 (fl, fr), *D. Espinel-Ortiz*, *C. Restrepo & A. Sanguano 269* (holotype: QCA (QCA-243282 and QCA-7010670 to QCA-7010679); isotypes: HA, HUTI, LOJA, Q, QCNE).

**Figure 6.**
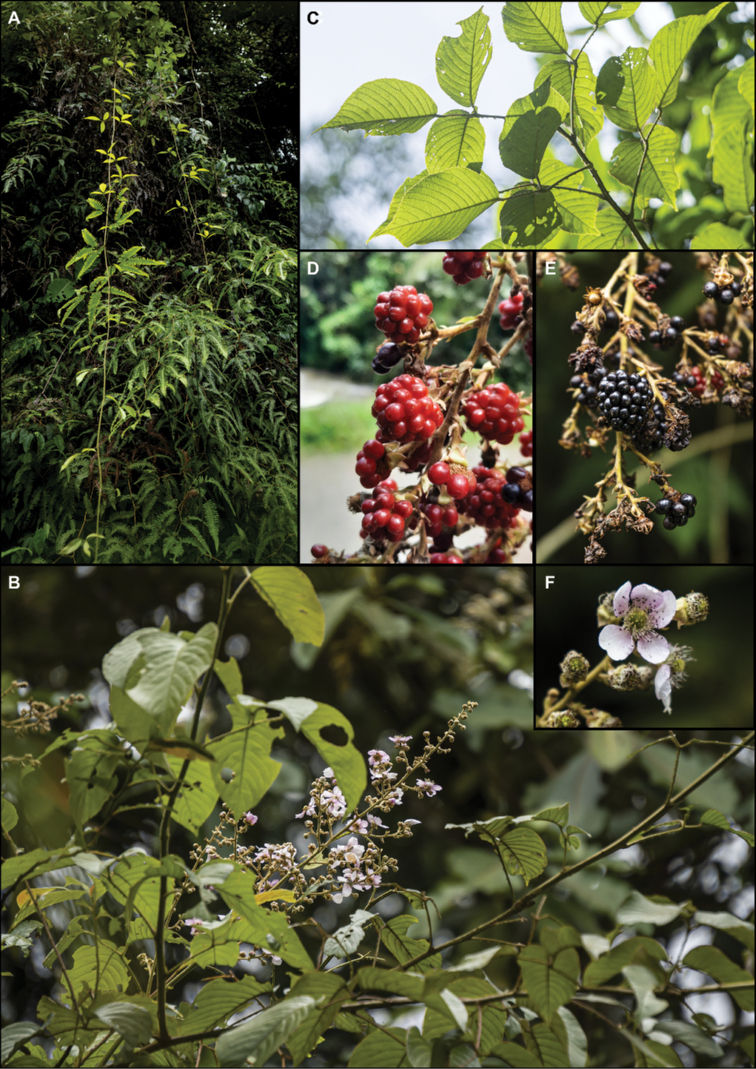
*Rubusmaquipucunensis* D.A. Espinel-Ortiz & Romol. **A** vine **B** scandent shrub and inflorescence **C** leaf abaxial surface **D** immature fruits **E** mature fruits **F** flower. Photos by Camilo Restrepo (**A–C, E–F**) and David A. Espinel-Ortiz (**D**).

#### Description.

**Woody vine** growing up to 20 m long, or **climbing shrub**. **Branches** obtuse-angled to slightly terete, woody, green to brown, densely villous to villous-hirsute, 2.0–12.1 mm diam., with scattered subsessile glands; unarmed or with 3–19 prickles (per total area of 5 cm long of the branch), gradually narrowed from a broad base, curved at the apex, 1.0–3.1 × 1.5–5.4 mm, glabrous. **Stipules** subulate, 3.9–9.2 × 0.1–0.3 mm, chartaceous, villous, with scattered sessile and subsessile glands. **Petioles** 3.8–10.4 cm long, villous, with (1–) 11–23 (–27) curved prickles 0.5–3.0 × 1.0–4.6 mm; lateral petiolules (3.6–) 9.1–13.8 mm long, unarmed or with up to 9 curved prickles 0.1–0.9 × 0.3–1.4 mm; terminal petiolules (2.3–) 3.6–5.3 cm long, with (4–) 18–35 curved prickles 1.0–2.0 × 0.8–3.6 mm. **Leaves** trifoliate; leaflets broadly elliptic or broadly ovate to elliptic, base rounded to obtuse or slightly subcordate, apex cuspidate to abruptly acute, margin serrate, lateral leaflets (5.4–) 7.5–12.5 (–17.1) × (3.4–) 4.1–9.2 (–12.2) cm, terminal leaflet (6.5–) 9.2–14.5 (–18.5) × (2.9–) 4.7–10.1 (–14.3) cm, chartaceous, with (7–) 11–16 (–18) secondary veins; adaxial surface villous-hirsute on primary and secondary veins with scattered short strigose hairs, or villous-hirsute in the midvein and sparsely adpressed villous in the veins; with subsessile and sessile glands, unarmed; abaxial surface sparsely villous and pilose on veins, or villous on veins and leaf blade with scattered subsessile glands, and (2–) 6–18 (–22) prickles on the primary vein, gradually narrowed from a broad base, straight to curved at the apex, 0.3–1.3 × 0.3–1.9 mm, glabrous. **Inflorescences** lax, compound, terminal cymes, 36–196-flowered, 22.6–59.4 cm long, with simple or trifoliate leaves below; peduncles terete, slightly light gold, 4.7–36.7 mm long, shortly lanate, with scattered sessile glands, unarmed or with 1–17 prickles, gradually narrowed from a broad base, straight to curved at the apex, 0.1–1.0 × 0.1–1.4 mm, glabrous; pedicels terete, slightly light gold, shortly lanate, 5.7–11.9 (–15.3) mm long, eglandular, unarmed. **Flowers** 14.2–22.6 mm diam.; sepals 5, broadly ovate to broadly elliptic, apex deeply mucronate, margin entire, 3.6–5.6 × 2.9–4.7 mm, tawny brown to ochre, acrescent; adaxial surface deeply concave, pannose, eglandular, unarmed; abaxial surface deeply convex, shortly lanate, and pannose on the margins and towards the apex, eglandular, unarmed; petals 5, broadly obovate, margin entire or erose, 5.6–11.6 × 5.2–10.1 mm, fuchsia when opening, completely pink or white with pink borders when fully opened, glabrous, eglandular, adaxial surface straight to concave, abaxial surface straight to convex; stamens with anthers glabrous, filaments fuchsia, glabrous; pistils, stigmas and styles glabrous, ovaries pilose. **Fruits** green to dark red when immature, and black at maturity, ovoid-globose, 11.0–14.8 × 12.1–15.6 mm (when fresh); drupelets 14–32 per receptacle, 4.0–6.1 × 3.1–5.4 mm (when fresh), pilose towards the base and apex.

**Figure 7.**
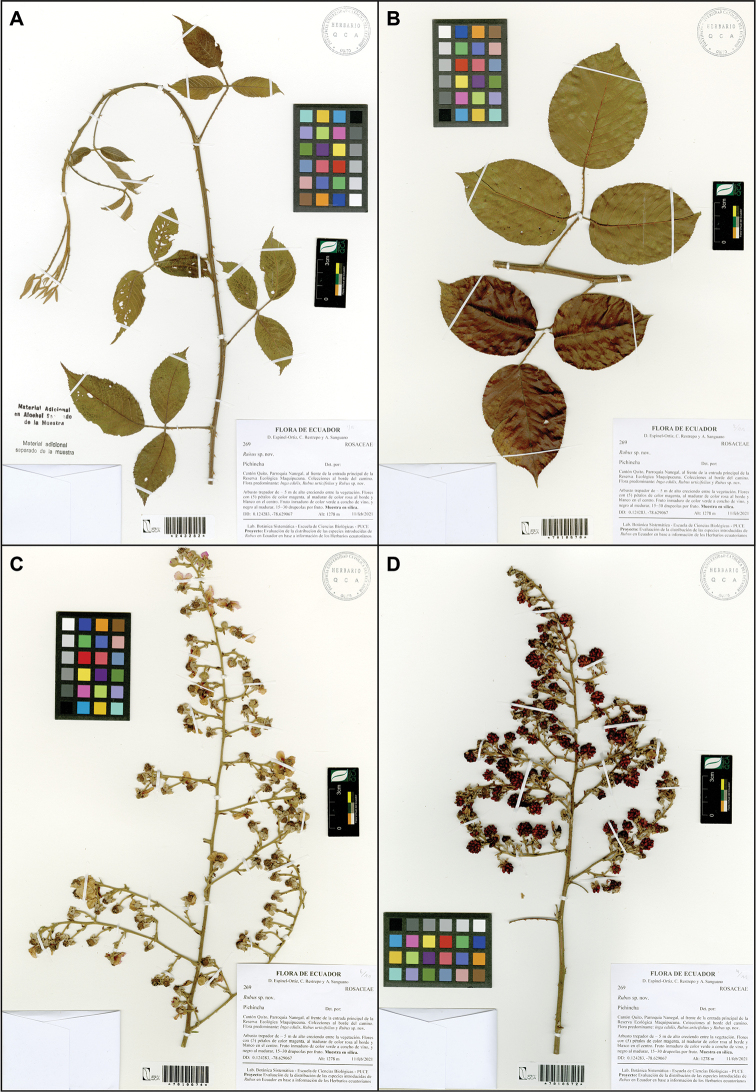
*Rubusmaquipucunensis* D.A. Espinel-Ortiz & Romol. Holotype collection D.A. Espinel-Ortiz, C. Restrepo & A. Sanguano 269 (QCA) **A** vine QCA243282 **B** climbing shrub QCA7010670 **C** inflorescence QCA7010674 **D** infructescence QCA7010672.

#### Additional specimens examined (Paratypes).

**Ecuador. – Santo Domingo de los Tsáchilas**: Old road along Chiriboga, Quito-Santo Domingo, 1275 m, 08 April 1984 (fl), *C.H. Dodson & M. Thurston 14195* (MO (MO-1559904)); old road San Juan-Chiriboga, km 60–70, 00°17.000'S, 78°50.000'W, 1000–1500 m, 09 Jan 1993 (fl), *K. Romoleroux & A. Freire 1514* (QCA (QCA-92036), QCNE (QCNE-77110)). – **Pichincha**: Near San Florencio, growing in subandes, 1889 (fl), *A. Sodiro 410*? (Q (Q-3613)); Ecological Reserve Maquipucuna, edge of pasture in secondary rainforest, trail from Hacienda El Carmen to Hacienda Esparragos, ca. 6 km airline SE of Nanegal, 00°07.500'N, 78°38.000'W, ca. 1300 m, 11 Sep 1989 (fl, fr), *G. Webster*, *K. Bainard & R. Schilling 27403* (DAV (DAV-331349 and DAV-331350), QCA (QCA-91821), QCNE (QCNE-44060)); Ecological Reserve Maquipucuna, secondary rainforest, trail from Hacienda Esparragos to Cerro de Sosa, ca. 5 km airline SE of Nanegal, 00°07.000'N, 78°38.000'W, 1400–1500 m, 18 Sep 1989 (fr), *G. Webster & M. Rios 27716* (DAV (DAV-331334), QCA (QCA-91761)); Ecological Reserve Maquipucuna, disturbed rainforest along Quebrada de la Cal, 4 km airline SE of Nanegal, 00°07.500'N, 78°38.000'W, 1250 m, 20 Jul 1990 (fl, fr), *G. Webster & B. Castro 28351* (DAV (DAV-331347 and DAV-331348), QCNE (QCNE-44101)); Ecological Reserve Maquipucuna, Maquipucuna mountains, Cerro Sosa, primary rainforest, 00°05.500'N, 78°37.000'W, 1725 m, 03 Jul 1991 (fl), *G. Webster*, *B. Castro & N. McCarten 28693* (DAV (DAV-331346)); Ecological Reserve Maquipucuna, trail S from Hacienda El Carmen, secondary rainforest, 00°07.000'N, 78°39.000'W, 1300 m, 06 Jul 1992 (fl), *G. Webster & UREP participants 29038* (DAV (DAV-331345), QCNE (QCNE-81119)); Ecological Reserve Maquipucuna, disturbed rainforest along trail from guava plantation to Alambí river, 00°07.300'N, 78°38.000'W, 1300–1400 m, 10 Jul 1992 (fl), *G. Webster & R. Rhode 29284* (DAV (DAV-331351), QCA (QCA-92244), QCNE (QCNE-75592)); same locality as for preceding, 1200–1400 m, 12 Jul 1992 (fl), *P. Delprete & G. Webster 6073* (QCA (QCA-240552)); Ecological Reserve Maquipucuna, trail to Cerro Montecristi, 00°07,070'N, 78°34,000W, 1700 m, 06 Nov 1999 (fl, fr), *C.E. Cerón*, *R. Arcos*, *C. Sevilla & A. Mosquera 39731* (QAP (QAP-28345)); Ecological Reserve Maquipucuna, disturbed rainforest along “Autoguiado’’ trail, 00°07.341'N, 78°37.741'W, 1258 m, 01 Sep 2020, *D. Espinel-Ortiz*, *E. Bastidas-León & C. Restrepo 239* (QCA (QCA-243392 and QCA-7010699)); same locality as for preceding, 00°07.294'N, 78°37.784'W, 1326 m, 11 Feb 2021 (fl), *D. Espinel-Ortiz*, *C. Restrepo & A. Sanguano 273* (HUTI, QCA (QCA-243371 and QCA-7010694)); Ecological Reserve Maquipucuna, trail to the river after orchid field, 00°07.449'N, 78°37.889'W, 1280 m, 11 Feb 2021, *D. Espinel-Ortiz*, *C. Restrepo & A. Sanguano 270* (QCA (QCA-243372 and QCA-7010695)); Ecological Reserve Maquipucuna, trail to the river, *00°07.350*'*N*, *78°38.158*'*W*, 1249 m, 11 Feb 2021, *D. Espinel-Ortiz*, *C. Restrepo & A. Sanguano 271* (QCA (QCA-243374)); same locality as for preceding, 00°07.419'N, 78°38.246'W, 1273 m, 11 Feb 2021, *D. Espinel-Ortiz*, *C. Restrepo & A. Sanguano 272* (HA, QCA (QCA-243373 and QCA-7010696)); Marianitas ca. 3 km after the bridge over river Alambí, road to Ecological Reserve Maquipucuna, 00°07.466'N, 78°38.810'W, 1239 m, 22 Feb 2021, *D. Espinel-Ortiz*, *C. Restrepo & A. Sanguano 275* (QCA (QCA-243375, QCA-7010697 and QCA-7010698)); same collection data as for holotype, 18 May 2021 (fl), *D. Espinel-Ortiz*, *Restrepo C. & O. Tejada 277* (QCA (QCA-243370)).

#### Distribution.

*Rubusmaquipucunensis* is distributed in the north of the Ecuadorian Western-Cordillera from 1000 to 1725 m a.s.l., in the provinces of Pichincha and Santo Domingo de los Tsáchilas (Fig. 4).

#### Ecology.

This species occurs in Chocó Andino rainforests dominated by trees, shrubs, and vines, and also in nearby disturbed areas. *Rubusmaquipucunensis* can be found living in sympatry with *Rubusurticifolius*. As branches grow older, they become glabrescent and lose prickles. Also, young leaves or leaves of juvenile individuals are significantly smaller and may seem different than the mature leaves. Flowering and fruiting branches grow at the top of the plant where more light is available, and it takes more than 15 days for the flowers to bloom. Flowering and fruiting collections dated from January, February, April, May, July, September and November.

#### Etymology.

The specific epithet honours the Ecological Reserve Maquipucuna (“Mano amiga” or “Friendly hand” in Kichwa) where a high number of samples were collected, and where this species is protected and easily found.

#### Preliminary assessment of conservation status.

*Rubusmaquipucunensis* is known from three localities of which two are impacted by human activity, including road opening, and the other locality is an Ecological Reserve. Following the IUCN (2019) guidelines, based on the reduced geographic distribution and altered land use, this species should be categorised as vulnerable (VU); at least until other populations are discovered.

#### Notes.

*Rubusmaquipucunensis* may resemble *R.boliviensis* by its habit and flowers, and *R.floribundus* by its habit and inflorescences, but differs from both species by its villous to villous-hirsute branches, in contrast with the pannose, pilose or puberulent to glabrescent branches of *R.boliviensis*, and tomentose to glabrescent branches of *R.floribundus*. Moreover, *R.maquipucunensis* has trifoliate leaves with broadly elliptic or broadly ovate to elliptic leaflets while *R.boliviensis* and *R.floribundus* have 5-foliolate leaves with ovate-elliptic leaflets. Furthermore, *R.maquipucunensis* has fruits with fewer (14–32) and bigger (4.0–6.1 × 3.1–5.4 mm) drupelets while *R.boliviensis* and *R.floribundus* have fruits with more (20–50 in *R.boliviensis*, and 40–50 in *R.floribundus*) and smaller (2.0–3.0 × 2.0–3.0 mm in *R.boliviensis*, and 2.5–4.0 × 2.0–3.0 in *R.floribundus*) drupelets. *Rubusmaquipucunensis* resembles *R.killipii* by its habit and long inflorescences, but differs by its shortly lanate peduncles and pedicels, and fuchsia to pink petals compared to the pannose peduncles and pedicels, and white petals of the latter. In addition, *R.maquipucunensis* has trifoliolate leaves while *R.killipii* has 5-foliolate leaves. As *R.killipii* fruits have not been described yet, they cannot be compared with those of *R.maquipucunensis*. *Rubusmaquipucunensis* resembles *R.selleanus* Helwig by its trifoliate leaves with broadly elliptic leaflets, but differs by its longer inflorescences (22.61–59.38 cm) compared to the shorter inflorescences (10–13 cm) of the latter. In addition, *R.maquipucunensis* has longer petioles (3.8–10.4 cm), bigger leaflets (7.5–12.5 × 4.1–9.2 cm) and sepals with mucronate apex, while *R.selleanus* has shorter petioles (1.5–3.5 cm), smaller leaflets (6–8 × 5.5–7 cm) and sepals with obtuse apex. Finally, *R.maquipucunensis* is found in Ecuador whereas *R.selleanus* has been recorded only in Hispaniola Island (Haiti and Dominican Republic).

##### ﻿Possible hybrids

### Rubus longistipularis × Rubus boliviensis

Taxon classificationPlantaeRosalesRosaceae

2869AA81-E196-59EB-BD65-FDFFB6F8BD1B

#### Specimens examined.

**Ecuador. – Imbabura**: Cotacachi, road Cuicocha-Apuela, Comuna Santa Rosa-Pucará, Apuela entrance, 00°21.826'N, 78°29.901'W, 1998 m, 28 Nov 2020, *D. Espinel-Ortiz*, *M.P. Ortiz*, *M.A. Espinel-Ortiz y C. Castillo 260* (QCA (QCA-243440, QCA-7010741 and QCA-7010742)). – **Pichincha**: Nono-Tandayapa road, between km 116–117, 00°01.787'S, 78°38.567'W, 1950 m, 02 Aug 2021, *D. Espinel-Ortiz*, *C. Restrepo & A. Sanguano 286* (HA, HUTI, QCA (QCA-243438 and QCA-7010738 to QCA-7010740)).

#### Notes.

The above specimens may be hybrids between *Rubuslongistipularis* and *R.boliviensis*. Both samples were collected in places where both species coexist and showed mixed characteristics from both species. For instance, the stipules of samples D. Espinel-Ortiz et al. 260 and D. Espinel-Ortiz et al. 286 were shorter (10.8–18.2 mm) and narrower (0.7–1.7 mm) than those of *R.longistipularis* (20.0–34.7 × 1.7–3.3 mm), but longer and wider if compared to those of *R.boliviensis* (6.0–10.0 × 1.0–2.0 mm). Furthermore, both samples showed sparsely pilose to pilose leaf margins and deeply villous to slightly pannose leaf abaxial surface, whereas *R.longistipularis* has tomentose leaf margins and pannose leaf abaxial surface, and *R.boliviensis* has glabrous leaf margin and densely villous leaf abaxial surface. The prickles from both samples were from the base ⅓–⅔ sparsely villous and glabrous towards the apex, while that of *R.longistipularis* is villous, and that of *R.boliviensis* is from the base ⅓ sparsely pilose to glabrous. Finally, sample D. Espinel-Ortiz et al. 286 showed mainly 3–5-foliolate leaves, while *R.longistipularis* has mainly trifoliate leaves and *R.boliviensis* has 3–5-foliolate leaves.

### ﻿Taxonomic key for Ecuadorian species

**Table:** 

1	Stipules linear-falcate, ovate or suborbicular; leaves simple or 3-foliolate (subg.Orobatus)	**2**
–	Stipules subulate or filiform; leaves 3-foliolate, palmately 5-foliolate or imparipinnate	**11**
2	Leaves simple	**3**
–	Leaves 3-foliolate	**7**
3	Stipules linear-falcate	***R.loxensis***
–	Stipules asymmetrically ovate	**4**
4	Upper leaf surface bullate	***R.azuayensis***
–	Upper leaf surface not bullate	**5**
5	Lower leaf surface pannose-tomentose	***R.acanthophyllos***
–	Lower leaf surface glabrous or sparsely pilose on veins	**6**
6	Flowers solitary or rarely in inflorescences 2–3 cm long, with less than 4 flowers	***R.coriaceus***
–	Inflorescences 5–9 cm long, with more than 5 flowers	***R.laegaardii***
7	Flowers solitary or in few-flowered lax inflorescences; sepals as long as or longer than petals	**8**
–	Flowers in simple or compound, compact inflorescences; sepals shorter than petals	**10**
8	Stipules ovate; flowers usually solitary or sometimes in inflorescences with 2–4 flowers	***R.glabratus***
–	Stipules suborbicular; inflorescences with more than 4 flowers	**9**
9	Lower leaflet surface glabrous or sparsely pilose, unarmed sepals	***R.roseus***
–	Lower leaflet surface tomentose or villous, prickly sepals	***R.nubigenus***
10	Leaves and inflorescences pubescent, prickly sepals	***R.nubigenus***
–	Leaves and inflorescences glabrous, unarmed sepals	***R.compactus***
11	Drupelets united and falling collectively from dry receptacle (subg. Idaeobatus)	**12**
–	Drupelets remaining on the fleshy receptacle and falling off together with it (subg. Rubus)	**14**
12	Leaves 3-foliolate; fruit yellow	***R.ellipticus***
–	Leaves imparipinnate, 5 or 7-foliolate; fruit pink-purplish to black or red	**13**
13	Lower leaf surface pannose with stipitate glands, whitish; stem pruinose	***R.niveus***
–	Lower leaf surface sparsely pilose with subsessile and sessile glands, greenish; stem not pruinose	***R.rosifolius***
14	Inflorescences few-flowered, usually less than 30 flowers per inflorescence (except for *R.maquipucunensis* and *R.longistipularis* that can have more than 40 flowers); basal leaves 3-foliolate, rarely 4 or 5-foliolate	**15**
–	Inflorescences many-flowered, usually more than 40 flowers per inflorescence; basal leaves 5-foliolate, rarely 3-foliolate	**21**
15	Stems glabrous, glaucous or puberulent	**16**
–	Stems tomentose, velutinous, villous, pilose or pannose	**17**
16	Stems glaucous; drupelets < 6 mm long, more than 50 per receptacle	***R.glaucus***
–	Stems not glaucous; drupelets > 7 mm long, less than 30 per receptacle	***R.megalococcus***
17	Leaflets with less than 9 pairs of secondary veins; stems pilose; petals greenish white	***R.adenothallus***
–	Leaflets with more than 10 pairs of secondary veins; stems tomentose, velutinous, villous or pannose; petals fuchsia, reddish violet, white or pink	**18**
18	Stems glaucous; stipules > 20 mm long; drupelets < 3 mm long, more than 50 per receptacle	***R.longistipularis***
–	Stems not glaucous; stipules < 14 mm long; drupelets > 4 mm long, less than 40 per receptacle	**19**
19	Vine or climbing shrub; leaflets broadly elliptic or broadly ovate to elliptic; up to 16 secondary veins	***R.maquipucunensis***
–	Scandent shrub; leaflets ovate to slightly elliptic; up to 13 secondary veins	**20**
20	Leaflet surface velutinous or tomentose, with sessile and subsessile glands	***R.bogotensis***
–	Leaflet surface sparsely villous or pilose, eglandular	***R.peruvianus***
21	Stems and branches glandular	**22**
–	Stems and branches eglandular	**24**
22	Stems densely covered with long-stipitate glands	***R.adenotrichos***
–	Stems with scattered, short-stipitate glands	**23**
23	Petioles pulvinate; base of the leaflets asymmetrical	***R.killipii***
–	Petioles not pulvinate; base of the leaflets rounded	***R.floribundus***
24	Lower leaflet surface glabrous; leaflets with 7–10 pairs of secondary veins	***R.killipii***
–	Lower leaflet surface pubescent; leaflets with 10–18 pairs of secondary veins	**25**
25	Stems with reddish, setose hairs	***R.urticifolius***
–	Stems tomentose, villous, pilose, pannose or glabrous, not setose	**26**
26	Leaflets with 10–12 or rarely 14 pairs of secondary veins, leaf-margins serrate	***R.floribundus***
–	Leaflets with 14–18 pairs of secondary veins, leaf-margins serrulate	***R.boliviensis***

## Supplementary Material

XML Treatment for Rubus longistipularis

XML Treatment for Rubus maquipucunensis

XML Treatment for Rubus longistipularis × Rubus boliviensis
